# Bayesian adaptive algorithms for locating HIV mobile testing services

**DOI:** 10.1186/s12916-018-1129-0

**Published:** 2018-09-03

**Authors:** Gregg S. Gonsalves, J. Tyler Copple, Tyler Johnson, A. David Paltiel, Joshua L. Warren

**Affiliations:** 1 Department of Epidemiology of Microbial Diseases, Yale School of Public Health, 60 College Street, New Haven, CT USA; 2 Independent Consultant, Yale School of Public Health, 60 College Street, New Haven, CT USA; 3 Department of Health Policy and Management, Yale School of Public Health, 60 College Street, New Haven, CT USA; 4 Department of Biostatistics, Yale School of Public Health, 60 College Street, New Haven, CT USA

## Abstract

**Background:**

We have previously conducted computer-based tournaments to compare the yield of alternative approaches to deploying mobile HIV testing services in settings where the prevalence of undetected infection may be characterized by ‘hotspots’. We report here on three refinements to our prior assessments and their implications for decision-making. Specifically, (1) enlarging the number of geographic zones; (2) including spatial correlation in the prevalence of undetected infection; and (3) evaluating a prospective search algorithm that accounts for such correlation.

**Methods:**

Building on our prior work, we used a simulation model to create a hypothetical city consisting of up to 100 contiguous geographic zones. Each zone was randomly assigned a prevalence of undetected HIV infection. We employed a user-defined weighting scheme to correlate infection levels between adjacent zones. Over 180 days, search algorithms selected a zone in which to conduct a fixed number of HIV tests. Algorithms were permitted to observe the results of their own prior testing activities and to use that information in choosing where to test in subsequent rounds. The algorithms were (1) Thompson sampling (TS), an adaptive Bayesian search strategy; (2) Besag York Mollié (BYM), a Bayesian hierarchical model; and (3) Clairvoyance, a benchmarking strategy with access to perfect information.

**Results:**

Over 250 tournament runs, BYM detected 65.3% (compared to 55.1% for TS) of the cases identified by Clairvoyance. BYM outperformed TS in all sensitivity analyses, except when there was a small number of zones (i.e., 16 zones in a 4 × 4 grid), wherein there was no significant difference in the yield of the two strategies. Though settings of no, low, medium, and high spatial correlation in the data were examined, differences in these levels did not have a significant effect on the relative performance of BYM versus TS.

**Conclusions:**

BYM narrowly outperformed TS in our simulation, suggesting that small improvements in yield can be achieved by accounting for spatial correlation. However, the comparative simplicity with which TS can be implemented makes a field evaluation critical to understanding the practical value of either of these algorithms as an alternative to existing approaches for deploying HIV testing resources.

**Electronic supplementary material:**

The online version of this article (10.1186/s12916-018-1129-0) contains supplementary material, which is available to authorized users.

## Background

Of the estimated 37 million people currently infected with the human immunodeficiency virus (HIV) worldwide, as many as 14 million remain unaware of their infection and unable to avail themselves of the antiretroviral therapy that could both prolong their lives and prevent the further spread of the virus to their sexual or needle-sharing partners [1]. Rates of undetected HIV infection are highly variable from one setting to the next, exceeding 60% in many parts of Africa, Eastern Europe, and the Middle East [2]. These sobering facts justify continued investigation of novel, cost-effective strategies to focus HIV screening efforts where they will maximize the yield of newly detected cases and to identify areas of concentrated recent infection (so-called HIV ‘hotspots’).

As we have described in previous work, the deployment of scarce resources to optimize the return on investment in HIV screening can be portrayed as an ‘explore-versus-exploit’ problem [3]. This canonical formulation, which emerges from the field of statistical decision theory, adopts the perspective of a decision-maker whose long-term objective is to maximize yield by making a sequence of short-term choices either to acquire better information about the prevailing state of a system (i.e., to explore) or to make the best possible decision based on the information already at hand (i.e., to exploit) [4, 5]. Under highly stylized conditions simulating a mobile HIV testing service, we have demonstrated that a simple, adaptive search algorithm consistently outperforms more traditional approaches used to deploy disease screening resources.

In this paper, we once again conduct a computer-based tournament to compare the performance of different approaches to targeted mobile HIV testing in a hypothetical city of geographic zones with differing rates of undetected HIV infection. As in our prior work [3], our aim is to understand the circumstances under which different search algorithms may or may not outperform one another. We report here on three important refinements to our prior assessment and their implications for decision-making. First, we have greatly enlarged the number of geographic zones considered. Second, we have admitted the possibility of spatial correlation in the prevalence of undetected HIV infection between adjacent zones. Finally, we have introduced and evaluated a new search algorithm that accounts for and capitalizes upon spatial correlation between zones.

## Methods

### Analytic overview

We used a computer simulation to compare the performance of three strategies for targeting mobile HIV testing services. We created a hypothetical city consisting of contiguous geographic zones, each with its own (unobserved) prevalence of undetected HIV infection.

Over each of 180 sequential rounds of play, hereafter referred to as days or days of testing, strategies were required to choose a single geographic zone in which to conduct a fixed number of HIV tests. Strategies were permitted to observe and remember the results of their own prior testing activities and to use that information in choosing where to test in subsequent rounds.

We define a ‘tournament run’ as a fixed number of sequential days. In the main analysis, all outcome measures used to evaluate the relative performance of one strategy against another are reported over a tournament run length of 180 days. Stable estimates of these performance measures and their variance are obtained by repeating each 180-day tournament run 250 times.

### HIV infection, hotspots, and spatial correlation

We constructed a hypothetical city consisting of geographic zones on a *n x n* grid. For the main analysis, consisting of the base case assumptions, we assumed that there were 36 zones (*i* ∈ {1, …, 36}) on a 6 *x* 6 grid. In sensitivity analyses considering alternative data simulation settings, we varied the total number of zones between 16 and 100.

The prevalence of undetected HIV infection, establishing the initial number of infected and uninfected persons, in a given zone was simulated using the following model:

$$ \mathrm{logit}\left({p}_i\right)={\beta}_0+{\phi}_i,\kern0.5em i=1,\dots, {n}^2 $$

where *p*_*i*_ is the prevalence for zone *i*, *β*_0_ is an intercept term that describes the center of the distribution of all prevalences, and *ϕ*_*i*_ is a value specific to zone *i* that determines how much zone *i*’s prevalence differs from the center of the distribution (large values indicate hotspots while lower values indicate cool spots or non-hotspots). For all data simulation settings, we fixed *β*_0_ to be − 5.00, centering the distribution of prevalences on 0.007. The *ϕ*_*i*_ values were simulated from a multivariate normal distribution, centered at zero, with a covariance matrix that allowed for the possibility of spatial correlation depending on the choice of an associated correlation parameter (large value indicates spatial independence while small value indicates high spatial correlation). Once the *ϕ*_*i*_ values were generated, we standardized them (the vector centered at zero with a standard deviation of one) in order to create a distribution of prevalence values with similar center/variability across all data simulation settings and, therefore, allowing us to more accurately attribute differences in the performance of each method to changes in the underlying data assumptions. We then multiplied each *ϕ*_*i*_ value by an inflation factor in order to create greater/fewer hotspots depending on the data simulation setting. Finally, once *ϕ*_*i*_ and *β*_0_ were selected, we calculated *p*_*i*_ for each zone using the inverse logit transformation and set all prevalences larger than 0.03 (the maximum hotspot value) equal to 0.03. Recognizing that not all persons with undetected HIV infection will be amenable to the offer of HIV testing, we capped the maximum prevalence of detectable HIV infection at 3%. This is slightly below the estimated prevalence of undetected HIV infection in high-risk African settings (e.g., Lusaka, Zambia). A new set of zone prevalences was generated using this framework for each of the 250 tournament runs of a given data simulation setting. Populations for each zone, *m*_*i*_, were drawn from a lognormal distribution based on the population of districts in the same representative African urban area (Lusaka, Zambia). Based on these final starting values for HIV prevalence of undetected HIV infection for each zone and the populations assigned initially to them, each zone thus began the simulation with a fixed number, rounded up to integer values, of infected (*p*_*i*_ × *m*_*i*_) and uninfected persons (*m*_*i*_ − [*p*_*i*_ × *m*_*i*_]) .

The main analysis was run over 180 days of testing and is meant to reflect the real-world potential use of these methods in the daily decision-making of HIV testing providers. We used the following notation to denote some useful population levels:

- *U*_*i*_*(t)*, the number of uninfected persons in zone *i* on day *t.* This was given by the sum of *OU*_*i*_*(t)* and *UU*_*i*_*(t)*, namely the number of observed and unobserved uninfected persons.
- *I*_*i*_*(t)*, the number of infected persons in zone *i* on day *t.* This was given by the sum of *OI*_*i*_*(t)* and *UI*_*i*_*(t)*, namely the number of observed and unobserved infected persons.
- $\frac{I{}_i(t)}{I_i(t)+{U}_i(t)}$, the prevalence of HIV infection in zone *i* on day *t*;
- *UP*_*i*_*(t)*, the prevalence of HIV infection among persons whose HIV infection status is unknown in zone *i* on day *t*. This was given by $\frac{UI_i(t)}{UI_i(t)+{UU}_i(t)}$
- *X*_*i*_(*t*), the number of previously undetected cases identified by screening in zone *i* on day *t.*

The yield of HIV testing, *X*_*i*_*(t)*, follows a binomial distribution with success probability *UP*_*i*_(*t*). Implicit in this formulation was the assumption that HIV tests are conducted only on persons with unknown HIV infection. In reality, a great deal of HIV testing takes place among persons whose infection status is already known. Our simplifying assumption could be relaxed to include repeat testing and to produce an across-the-board reduction in the effectiveness of screening; however, this would have no impact on the relative yield of different strategies (our performance measure of interest). We also assumed that the population in a given zone greatly exceeds the number of HIV tests that can be performed in that zone in a single day. This permitted us to make the additional simplifying assumption that sampling for HIV on any given day occurs ‘with replacement’. This assumption too could be relaxed without overly complicating the analysis but would not likely have a material impact on the performance results of interest.

At the end of each day, the prevalence of HIV infection among persons whose status is unknown, *UP*_*i*_(*t*), was updated to account for three different considerations. First, ‘shelf life’, where the reliability and relevance of a negative result declines with the passage of time. We assumed that observed uninfected individuals eventually return to the pool of unobserved uninfected individuals. Second, ‘new arrivals’, where, as described above, we permitted the arrival of persons with unobserved HIV infection status (both infected and uninfected). Finally, ‘new HIV testing’, through which, if *m* HIV tests were conducted in zone *i* on day *t*, the unknown prevalence the following day was updated as follows:

$$ {UP}_i\left(t+1\right)=\frac{UI_i(t)-{X}_i(t)}{\left[{UI}_i(t)-{X}_i(t)\right]+\left[{UU}_i(t)-\left(m-{X}_i(t)\right)\right]}. $$

### Strategy 1: Thompson sampling (TS)

TS is an adaptive algorithm whose actions aim to maximize expected value based on random sampling from prior probability distributions on the prevalence of undetected HIV in each zone. These prior distributions are themselves the ex post result of updates based on previous rounds of observation. The user seeds the algorithm with initial probability distributions for the prevalence of undetected HIV in each zone at time 0. At the start of each day, TS samples randomly from its current probability distribution for each zone. It then elects to conduct testing in whichever zone yields the largest realized value (note that the zone selection process is based on random sampling from prior probability distributions – the algorithm’s ‘belief structure’ – and not from any actual HIV testing in a zone; this indirect selection mechanism ensures that every zone has a non-zero probability of being chosen for testing on any given day while, at the same time, ensuring that a zone will be selected with a probability that is proportional to the strength of the algorithm’s beliefs about how much undetected HIV infection exists in that zone). If a zone is selected for testing on a given day, the results of those testing activities will be employed to update the algorithm’s prior beliefs for that zone; the posterior distribution that results from that updating process will become the sampling distribution for zone selection on the subsequent day.

We used a *Beta*(*α*_*i*_*, β*_*i*_) distribution to describe TS’s beliefs about the prevalence of undetected HIV infection in zone *i*. The *Beta*, a continuous distribution on the interval (0, 1), is a natural choice for this purpose; first, because it is conjugate to the binomial distribution (i.e., a *Beta* prior and *Binomial* likelihood will yield a *Beta* posterior) and, second, because its two parameters are easily interpreted as ‘total observed positive HIV tests’ and ‘total observed negative HIV tests’, respectively. Thus, if *m* new HIV tests yield *x* new cases detected in zone *i*, the posterior probability will follow a *Beta*(α_i_ + *x*, β_i_ + (*m*–*x*)) distribution (see Additional file 1 for more details).

### Strategy 2: Besag York Mollié model (BYM)

Conditional autoregressive (CAR) models are used to account for spatial correlation in areal data when what is observed in neighboring regions is assumed to be more similar than observations occurring at larger distances [6]. They can be incorporated into Bayesian hierarchical models and the Besag York Mollié (BYM) framework used here employs an intrinsic CAR (ICAR) distribution (improper version of the CAR model) for the spatial random effects and exchangeable, normally distributed random effects to account for non-spatial heterogeneity in the data [7].

Similar to TS, our BYM modeling strategy begins the sampling process by assuming independent *Beta*(*α*_*i*_*, β*_*i*_) prior distributions for the prevalence of undetected HIV infection in each of the zones. During an initial ‘learning’ period, the BYM model proceeds in the same way as TS, selecting a zone for testing on a given day by sampling from its current probability distribution for each zone’s prevalence of undetected HIV prevalence and then choosing the zone that yields the largest realized value. Using TS, when the number of completed days is low, zones are selected almost at random. This is because TS assumes an uninformative, independent *Beta*(1, 1) prior distribution for the prevalence of undetected HIV infection in each zone and little new information across all zones is collected at the beginning of the simulations. As a result, on average, we observe a mix of low and high prevalence zones that are used to fit the BYM model for the first time. At the end of the learning period, the BYM model is fitted to the total set of collected data from each individual zone (number of identified infected individuals versus total number of sampled individuals in each zone). The choice of 10 zones for the initial learning period was made to ensure we had a reasonable number of spatial data points with which to fit the BYM model. For instance, it would be impossible to learn about the spatial correlation in the data using only data from a single spatial region. Once the BYM model is fitted to the current set of observed data, the marginal posterior predictive distribution of the underlying prevalence of undetected HIV cases at each zone is obtained via Markov chain Monte Carlo (MCMC) posterior sampling. We then randomly select a single value from each of these zone-specific distributions and identify the zone that corresponds to the largest value. This zone is selected for sampling on the subsequent day. This process is then repeated until the end of the simulation time period.

Unlike TS, which only gathers information as it visits a given individual zone, the BYM model can leverage inter-zone correlation to take what it observes in one zone and use that information to draw useful inferences about the prevalence of undetected HIV in neighboring zones. The model for the underlying prevalence at each zone is a function of a shared intercept, a spatially correlated random effect (ICAR distribution), and an exchangeable, normally distributed random effect (logistic regression model assumed). Because the intercept is shared across all zones, as data are gathered about a particular zone, the model is simultaneously learning about the value of the intercept and, therefore, about all zone prevalences. Similarly, because the spatial random effect assumes similarity between neighboring zones a priori, as data are gathered on a particular zone, the model is also learning about that zone’s neighbors (and beyond). The exchangeable random effect ensures that all variability in the prevalences is not attributed to spatial similarity and therefore prevents the model from oversmoothing the data. In the case of no spatially correlated variability and complete independence between data from the different zones, the BYM model will collapse to something very similar to TS (see Additional file 1 for more details).

### Strategy 3: Clairvoyance

For purposes of benchmarking, we sought to establish a credible upper-bound on the number of new HIV cases that any search strategy could possibly detect. To that end, we developed the Clairvoyance strategy, an algorithm that chooses to test in whichever zone has the greatest underlying prevalence of undetected HIV infection on any given day. Clairvoyance has access to perfect current information about new arrivals/departures, about individuals whose previous test results have exceeded their shelf life, and about the results of its previous testing activities. This permits it to select the most promising zone for testing on any given day. We emphasize, however, that it has no special knowledge about the HIV-infection status of any individuals selected for testing within that zone. Like any other strategy, it samples with replacement within whichever zone it selects.

### Parameter estimates, main analysis, and sensitivity analyses

Initial parameter values as well as those used in the sensitivity analyses are described in Tables 1 and 2. Our goal was to understand the performance of strategies under a broad variety of plausible data simulation settings. We therefore defined parameter ranges that reflected observations drawn from a multiplicity of international settings. Areas differ in terms of population size. Numbers of infected and uninfected persons in a zone were assigned via random realizations from a lognormal distribution (rounded to the nearest integer) that was itself estimated using 2010 census data on the number of adults aged 15–59 years living in urban wards of Lusaka, Zambia [8]. We explored values ranging from less than 0.5% to 3.0%, for the underlying prevalence of undetected HIV infection, reflecting zones with lower numbers of undetected individuals and zones that can be considered hotspots. The prevalence of undiagnosed HIV infection in some settings, including sub-Saharan Africa, can be larger than 3%. For instance, 12.3% of Zambian adults (15–59) are HIV positive, but 32.7% of them do not know their serostatus, and thus 4% of adults are still undiagnosed [9, 10]. However, we chose the 3% ceiling of undetected HIV prevalence in this simulation to represent a fraction of this population, as not all undiagnosed individuals will necessarily come forward for testing.

**Table 1:** Parameter main analysis values

Parameters	Values
Overall population	Simulate from lognormal distribution based on 2010 Lusaka, Zambia census
Grid dimensions	6 × 6
Level of correlation, percentage of hotspots in grid	Low, 20% (on average)
Percentage of new infections (times zone population divided by 365 days)	0.66%
Percentage of new HIV-negative arrivals (times zone population divided 365 days)	3.4%
Days until return to unobserved, uninfected pool	45
Initial observed HIV^+^/HIV^–^ (priors for TS)	*Beta*(1, 1)
Initial observed HIV^+^/HIV^–^ (priors for ICAR/BYM during learning period)	*Beta*(1, 1)
Intercept (priors for ICAR/BYM)	*Normal*(0, 2.85)
Priors for ICAR and exchangeable random effects	*Inverse-Gamma*(3, 2)
Days of testing	180
Tests per day	25

*BYM* Besag York Mollié, *ICAR* intrinsic conditional autoregressive, *TS* Thompson sampling

**Table 2:** Parameter values for sensitivity analysis

Parameters	Values
Grid dimensions	4 × 4; 5 × 5; 10 × 10
Percentage of new HIV-negative arrivals (times zone population divided 365 days)	0, 1.7%, 6.8%
Percentage of new infections (times zone population divided by 365 days)	0, 0.33%, 1.32%
No arrivals or infections	0
Level of correlation, percentage of hotspots in grid	None, 20% (on average)
Level of correlation, percentage of hotspots in grid	Low, 10% (on average)
Level of correlation, percentage of hotspots in grid	Low, 30% (on average)
Level of correlation, percentage of hotspots in grid	Medium, 20% (on average)
Level of correlation, percentage of hotspots in grid	High, 20% (on average)
Days until return to unobserved, uninfected pool	10, 90
Days of testing	90, 365
Tests per day	10, 40

We considered different rates of population movement, setting in-migration of new HIV-negative individuals at an annual 3.4% of a zone’s population in the main analysis, so that the daily number of new HIV-negative individuals entering a zone was 3.4% times the zone’s population divided by 365 days. The main analysis data simulation setting was derived from projections from the 2010 Zambian census for Lusaka [8]. In the sensitivity analyses, we doubled this number in each zone to reflect fast-growing settings but we also considered a case with half of the base case values and with no in-migration in sensitivity analyses. In the main analysis, zones were assigned HIV incidence rates based on annual incidence rates for Lusaka and daily new infections took the annual incidence figure (0.66%), multiplied it by the population of each zone and divided it by 365 days [9]. In the sensitivity analyses, we doubled this figure to represent faster growing epidemics, and also considered a case with half of the base case values and with no new infections. Finally, we also examined the case where no new HIV-negative and no new HIV infections occurred daily in each zone.

Other HIV testing program parameter ranges were selected to correspond roughly to values reported in the literature. We relied on two South African studies to assume that a mobile testing service could conduct *m* = 25 tests in a given zone on a given day; daily values ranging from 10 to 40 tests were considered in sensitivity analyses [11, 12]. We further assumed that individuals who are found to be uninfected return to the unobserved uninfected pool after 45 days, with values ranging from 10 to 90 days in the sensitivity analysis [13, 14]. Finally, we conducted the main analysis over 180 days (sensitivity analyses range, 90–365 days), reflecting our assumption that decision-makers might devote a half year to experimenting with new approaches to deploying HIV testing resources.

In the main analysis, the spatial correlation was set in the ‘low’ setting, where we defined ‘low’ as the correlation between prevalences from the two closest zones (i.e., based on distance between zone centroids) equal to 0.20. Spatial correlation was defined as a function of distance between zone centroids, with increasing distance leading to decreasing correlation. In subsequent sensitivity analyses, we varied the spatial correlation as follows:

1. None: Maximum correlation capped at 1 × 10^–100^ (independence);
2. Low: Maximum correlation capped at 0.20;
3. Medium: Maximum correlation capped at 0.50;
4. High: Maximum correlation capped at 0.90.

In addition, for the main analysis, we scaled the *ϕ*_*i*_ value by 1.80 (on average 20% of the zones were hotspots) while, for sensitivity analyses, we increased this value to 2.90 (30% hotspots) to create more extreme prevalence values and decreased it to 1.20 (10% hotspots) to create less variability (i.e., fewer hotspots) in the distribution of prevalences across all zones.

Both the TS and BYM strategies require the user to specify their ‘initial beliefs’ – that is, the probability distributions for the prevalence of undetected HIV infection in each zone at *t* = 0. For TS, we applied uniform(0, 1), uninformative *Beta*(1, 1) distributions to all zones. This reflected the highly conservative assumptions that virtually nothing is known about the starting prevalence of HIV infection in any of the zones. For the BYM strategy, we also assumed *Beta*(1, 1) prior distributions for the zone prevalences at the outset of the learning period. The intercept term was given a *N*(0, 2.85) prior distribution while the variance parameters associated with the ICAR and exchangeable random effects were each assigned inverse-gamma(3, 2) prior distributions. The prior distribution for the intercept resulted in an approximately uniform(0, 1) prior distribution for zone prevalences under the assumption of no additional variability.

To ensure we could statistically differentiate the performance of each of the methods, the tournament was run 250 times for each of the data simulation settings. Performance statistics reported in the Results section below represent averages across these 250 tournament runs as well as an examination of the absolute number of new diagnoses (minimum, first quartile, median, third quartile, and maximum) detected during these 250 tournament runs by each strategy. A strategy was deemed to have outperformed another in a head-to-head comparison if it detected a greater number of new cases in at least 55.25% of the 250 tournament runs. This significance value represents the threshold for a difference in proportions with *p* < 0.05 in a one-sided Z-test. We also examined the difference in the mean number of cases detected by each strategy, assessing significance with a one-side Welch’s *t* test.

## Results

### Main analysis

**Fig. 1:**
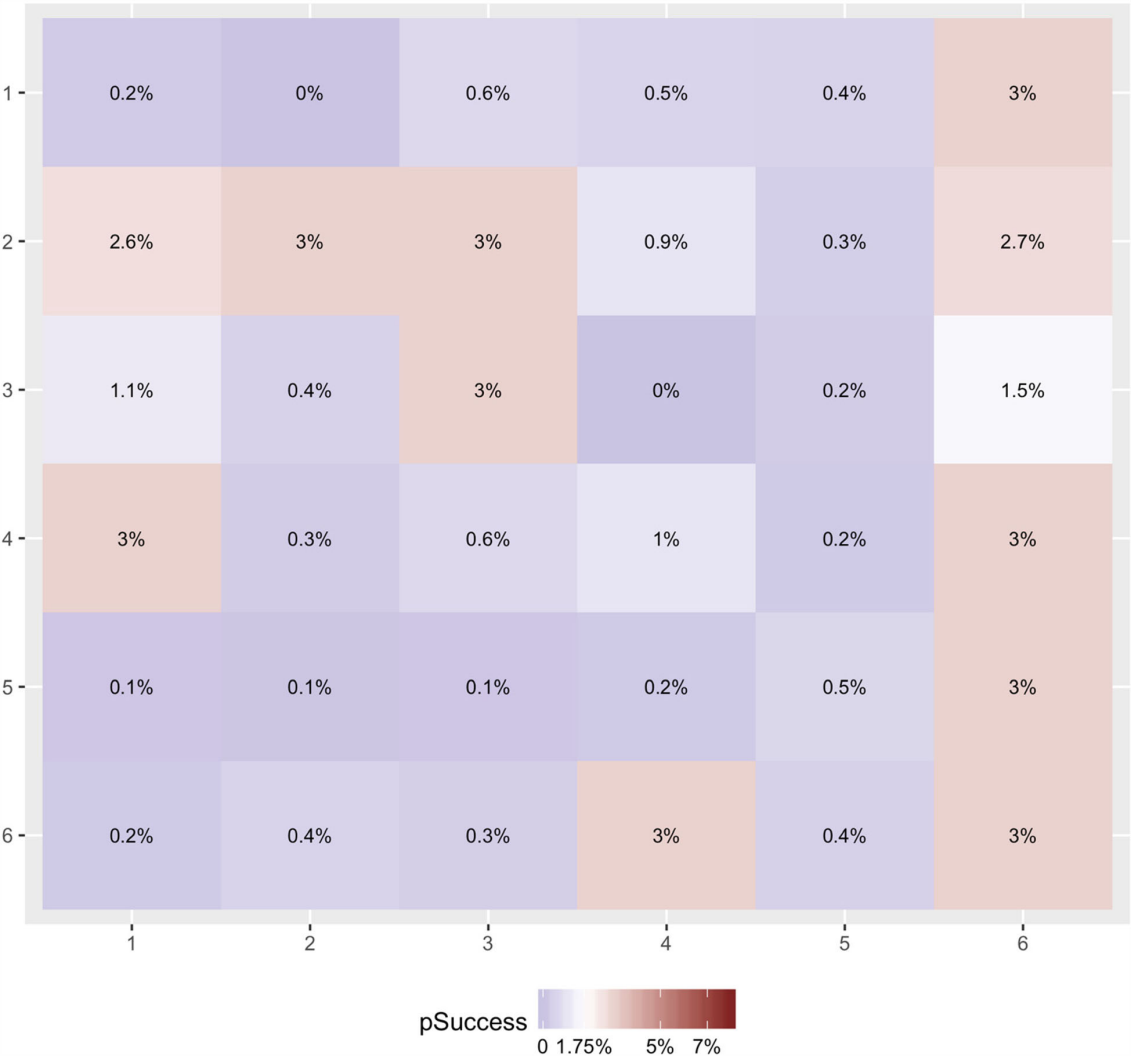
Example of grid of true underlying prevalences of undiagnosed HIV infection. The values in the individual squares represent the starting value (*t* = 0) of prevalences of undiagnosed HIV infection (UP, (t)) for each zone. Each iteration of a given data simulation setting starts with a new formulation of this grid and this is a representative sample of a grid for the base case with low correlation and 20% hotspots on average (although this single example from the base case has 30% hotspots). All strategies start with the same grid in any given iteration

**Fig. 2:**
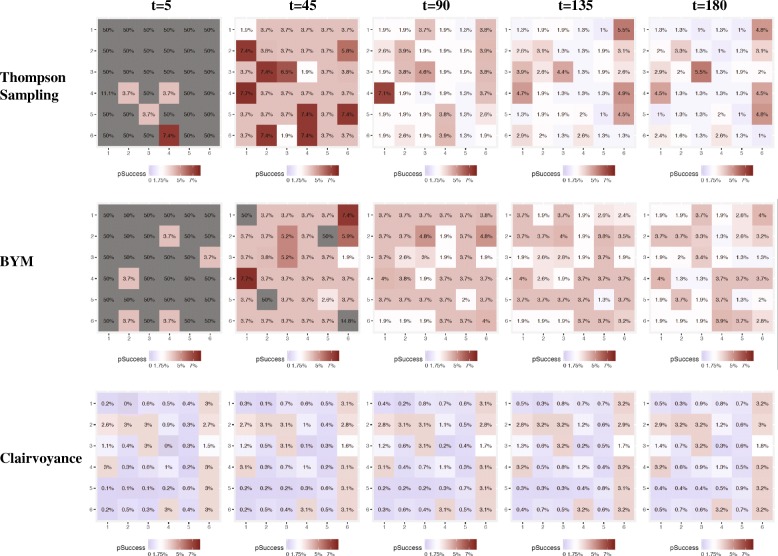
Estimated prevalence of undiagnosed HIV infection by strategy at five time points. Each strategy, except for clairvoyance, which knows the true underlying probability of undiagnosed HIV infection at all times, updates its estimates of each zone’s prevalence during the course of the simulation as it gathers new information. This is a representative set of grids for the estimates made by the three strategies at *t* = 5, 45, 90, 135, and 180 days

**Fig. 3:**
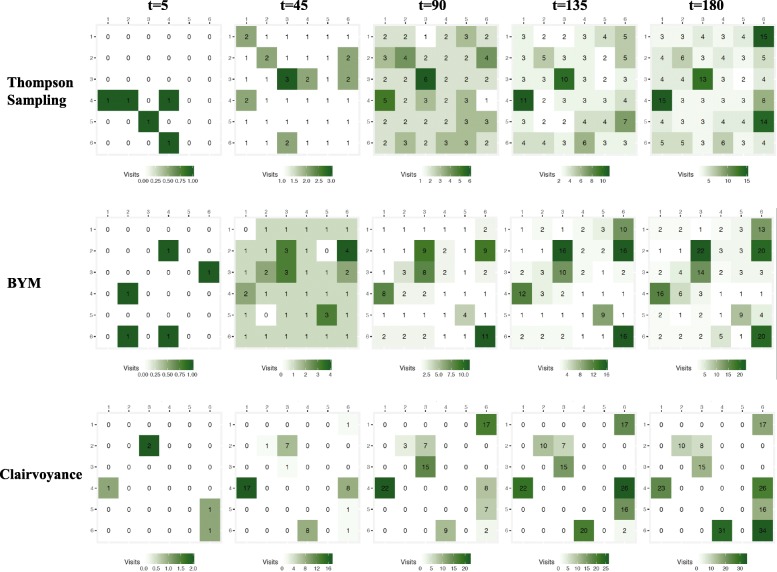
Cumulative visits to each zone by strategy at five time points. Each strategy, over the course of the simulation, visits multiple zones as it attempts to converge on hotspots of undiagnosed HIV infection. This is a representative set of grids for cumulative visits made to each zone by the three strategies at *t* = 5, 45, 90, 135, and 180 days

Figure 1 shows a representative 6 × 6 grid from the main analysis, consisting of 36 zones with low spatial correlation in the data and with 30% of the zones being hotspots. Across the 250 tournament runs, the average proportion of hotspots was roughly 20%. While a new grid of prevalences for the zones is generated for each set of 250 tournament runs of a given data simulation setting, Fig. 1 is meant to offer an example of what the underlying structure of probabilities looks like at *t* = 0 before the 180 days of testing begin. In each of the 250 tournament runs, all strategies begin with the same underlying grid of prevalences. Figure 2 shows the estimated prevalence of undetected HIV infection assumed by each strategy in the main analysis at five time-points (*t* = 5, 45, 90, 135, and 180 days). Figure 2 shows that the TS and BYM estimates of the underlying prevalence of undetected HIV infection shifts over time but in different ways. BYM’s estimation of the underlying prevalence of undetected HIV infection among the zones declines over time, but the algorithm maintains estimates that are higher than those of TS across all 180 days of testing in more zones than TS. With TS, estimates of prevalences among the zones, particularly non-hotspots, declines earlier on. This can be seen in the shift from reds to blues in the top panel (TS) of Fig. 2 in contrast to the middle panel where reds still predominate (BYM) as the number of days of testing in the simulation mounts. The Clairvoyance strategy in Fig. 2 has perfect information on the prevalence of undetected HIV infection on each day and thus its ‘estimate’ represents the actual values on the grid and impact over time of new, incident HIV infections, new HIV-negative in-migration, the ‘shelf life’ of HIV-negative test results, and its own success at finding new cases of HIV infection. Figure 3 shows the aggregate visits to each zone up until each of the same five time-points for all strategies. BYM visits and exploits hotspots more often than TS over time (conversely spending less time in non-hotspots than TS), while TS continues to explore more zones, even those that are non-hotspots, over the course of the 180 days. Clairvoyance visits all the hotspots of 3.00% prevalence in rotation throughout the 180 days and spends no time elsewhere. Clairvoyance does not even visit hotspots with slightly lower prevalence values (e.g., 2.70%). We provide versions of these three figures for data simulation settings with medium and high spatial correlation as additional files for readers interested in seeing the performance of the three strategies under these conditions (Additional file 2: Figure S1–S6).

**Fig. 4:**
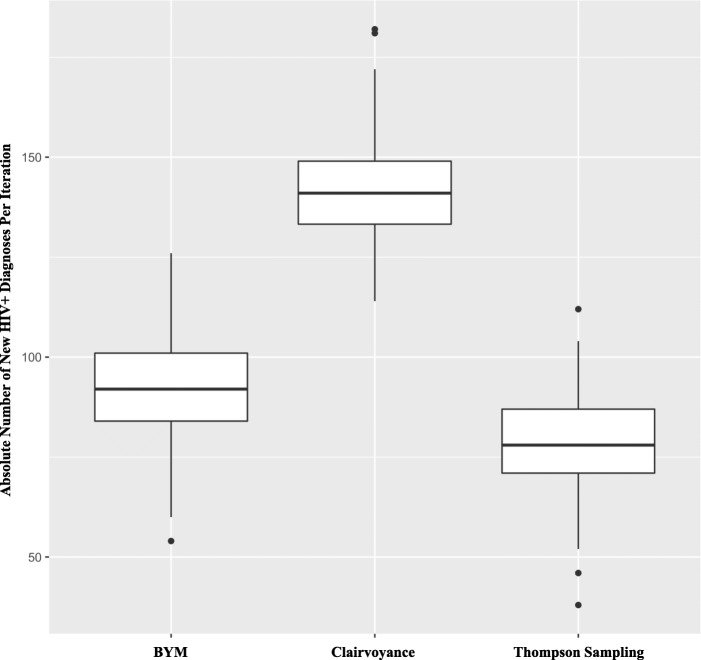
Basic statistics for yield of new HIV diagnoses by strategy. The minimum, first quartile, median, third quartile, and maximum number of new diagnoses detected by each strategy over 180 days in 250 iterations of the simulation for the main analysis

Figure 4 shows the key results for the main analysis, indicating the absolute number of new diagnoses detected by each strategy over 180 days (minimum, first quartile, median, third quartile, and maximum) in 250 tournament runs of the simulation. Clairvoyance outperformed all other strategies in overall mean number of new HIV diagnoses detected, identifying 141.87 (SD 11.83) new cases over the course of the 250 tournament runs, while TS uncovered 78.24 (SD 11.44) and BYM found 92.59 (SD 12.37). These results are also shown in Table 3 and Additional file 3: Table S1. The differences in the mean number of cases detected over 250 tournament runs between TS and BYM, TS and Clairvoyance, and BYM and Clairvoyance were all significant by Welch’s *t* test (*p* < 0.0001). This indicates that TS and BYM identified 55.1% and 65.3%, respectively, of the total infections detected by the Clairvoyance strategy. Finally, over the course of 250 tournament runs in the main analysis in pairwise head-to-head competition, BYM won 80% of the time over TS, with Clairvoyance winning 100% of the time against TS and BYM. These results are significant by a one-sided Z-test of a difference in proportions (*p* < 0.0001).

**Table 3:** Results for main analysis and sensitivity analyses

Parameters	Values	Thompson sampling			Besag York Mollié		
		Win percentage	New diagnoses	New diagnoses	Win percentage	New diagnoses	New diagnoses
			Mean	Std Dev		Mean	Std dev
Main analysis	N/A	20%	78.24	11.44	80%	92.59	12.37
percentage of new HIV-negative arrivals (times zone population divided 365 days)	0	17%	79.75	12.02	83%	96.40	11.50
percentage of new HIV-negative arrivals (times zone population divided 365 days)	1.7%	19%	79.07	11.21	81%	92.88	11.52
percentage of new HIV-negative arrivals (times zone population divided 365 days)	6.8%	16%	77.53	12.10	84%	93.17	10.85
Grid dimensions	4 × 4	54%	94.94	13.73	46%	93.18	13.85
Grid dimensions	5 × 5	38%	85.70	13.30	62%	91.98	11.84
Grid dimension	10 × 10	28%	65.81	9.52	72%	74.69	11.40
Percentage of new infections (times zone population divided by 365 days)	0	13%	71.99	11.19	87%	88.34	11.40
Percentage of new infections (times zone population divided by 365 days)	0.33%	14%	74.52	10.72	86%	89.88	10.21
Percentage of new infections (times zone population divided by 365 days)	1.32%	20%	85.46	11.30	80%	99.84	12.63
No arrivals or infections	N/A	20%	78.24	11.44	80%	92.59	12.37
Level of correlation, percentage of hotspots in grid	None, 20% (on average)	18%	78.20	12.07	82%	93.97	12.57
Level of correlation, percentage of hotspots in grid	Low, 10% (on average)	16%	68.13	9.70	84%	82.93	13.29
Level of correlation, percentage of hotspots in grid	Low, 30% (on average)	23%	88.59	11.89	77%	100.56	11.82
Level of correlation, percentage of hotspots in grid	Medium, 20% (on average)	18%	78.83	11.31	82%	93.62	11.86
Level of correlation, percentage of hotspots in grid	High, 20% (on average)	14%	78.41	11.56	86%	95.86	11.38
Days until return to unobserved, uninfected pool	10	23%	79.46	12.34	77%	92.54	12.00
Days until return to unobserved, uninfected pool	90	16%	78.28	10.90	84%	93.58	12.05
Days of testing	90	27%	33.17	6.84	73%	39.81	7.83
Days of testing	365	30%	193.79	20.24	70%	206.10	21.31
Tests per day	10	27%	27.55	6.07	73%	32.53	6.83
Tests per day	40	29%	137.54	15.17	71%	148.91	16.43

### Sensitivity analyses

We re-evaluated all findings using the settings specified in Tables 1 and 2. The mean number (and SD) of new diagnoses detected by TS and BYM in the main analysis and in all sensitivity analyses are described in Table 3. Under every scenario we examined in sensitivity analysis, Clairvoyance detected the greatest number of new HIV-positive cases (see Additional file 3: Table S1 for mean number of new diagnoses detected by Clairvoyance in the main analysis and in all sensitivity analyses). BYM almost always outperformed TS. TS narrowly defeated BYM when we considered a smaller grid size (e.g., 4 × 4) but this margin of victory (i.e., differences in the number of new diagnoses) was not statistically significant.

Sensitivity analysis revealed that the margin of victory between TS and BYM remains small under almost all circumstances. Averaging across all sensitivity analyses, the difference in the number of cases detected between TS and BYM was just over 12 cases. By contrast, Clairvoyance’s average margin of victory over its competitors exceeded 50 cases.

## Discussion

In our previous work, we introduced TS as a potential method for more efficiently deploying mobile HIV testing services and suggested that this algorithm could be useful in improving the detection and diagnosis of other infectious or chronic diseases [3]. In that study, TS was pitted against, and consistently outperformed, a winner-take-all strategy that sampled each geographic zone consecutively before deciding, based upon the zone with the largest yield of new diagnoses, where to devote all of its remaining testing resources. This winner-take-all strategy will not work for a larger collection of zones since a stepwise approach is time-consuming, with initial sampling periods quickly exceeding number of days of testing in the simulation. Thus, we were interested in finding other algorithms that could be compared against TS in an expanded setting and in particular where spatial correlation may exist in terms of the probability of finding new cases of undetected HIV infection in neighboring zones.

While BYM is a widely used method in spatial statistics and epidemiology, used to map disease occurrence and to predict outbreaks, it has not generally been deployed in public health as a spatial sequential decision-making tool and we can consider this a novel potential use for it [15, 16]. In other settings, particularly environmental management and commercial applications such as oil exploration, related methods have been used to model space-structured sequential decision-making under uncertainty [17–19].

The BYM model deployed here represents an improvement on the yield of new diagnoses over TS in our tournament. In almost all cases it outperforms TS, except when the number of zones is smaller (i.e., when the grid size is 4 × 4). This is not surprising as during the BYM model’s learning period (up until 10 zones), the algorithm is following the same procedural steps as TS. With 16 zones, BYM has only just begun to incorporate information about neighboring zones into its decision-making process.

What is surprising is that, while BYM outperforms TS in all other settings, there does not seem to be an advantage for BYM in settings with higher spatial correlation in the data. This may be because the number of zones considered in this work is too small to fully exploit the benefits of modeling the spatial correlation. In cases where there is a larger number of zones and fewer hotspots, it may be more important to model the spatial correlation to avoid spending excess time in low prevalence areas. However, BYM’s stronger performance overall may be due to the fact that BYM continues to incorporate information across zones during estimation even in the absence of spatial correlation. The intercept parameter and exchangeable random effect variance parameter are shared across all zones. This should allow the BYM model to quickly learn about low prevalence areas and avoid spending time in them. In fact, this is demonstrated in Fig. 3, as BYM makes fewer visits to lower prevalence areas than TS.

There are several implications of these findings. First, the BYM model in simulation is a better tool for detecting new cases of undetected HIV infection in most settings than TS. Second, because it is difficult to make assumptions about whether there is indeed correlation in the data (is the probability of finding new cases of undetected HIV infection from one zone to another linked neighbor-to-neighbor?) there is a strong rationale to rely on BYM as it is functionally similar to TS in the absence of spatial variability.

However, there are operational complexities with BYM that might make it less attractive as a tool for use in the field. TS is a simple algorithm that can be implemented in a spreadsheet with a few formulas and requires only a daily report of new HIV-positive and HIV-negative diagnoses for the Bayesian updating process. By contrast, the BYM model can be computationally demanding in comparison to TS (depending on the number of zones) because of its reliance on MCMC model fitting techniques; the convergence of the MCMC algorithm must be assessed, it requires the ability to determine the neighborhood structure of the data (e.g., shapefiles for different regions are needed) and a certain number of zones need to be visited before estimation stabilizes [20, 21]. Integrated Nested Laplace Approximation often represents a computationally convenient alternative to Bayesian model fitting and provides approximations to marginal posterior distributions for model parameters. It can also be used to fit the BYM model if MCMC techniques become computationally difficult due to an extremely large number of zones in a particular application. However, both MCMC and Integrated Nested Laplace Approximation still remain more complex to utilize than TS, which can be implemented using a spreadsheet program or by hand [22, 23]. While BYM performs better than TS in simulation, its modest margin of victory (~ 10%) in yield of new infections diagnosed must be weighed against these practical difficulties. In resource-poor settings (in fact, any settings without sufficient computing infrastructure and statistical support) the logistical simplicity of implementation might commend TS as the preferred tool for locating HIV testing services.

Because TS and BYM only detected 55.1% and 65.3%, respectively, of the total infections detected by Clairvoyance there may be room for improvement in the yield of new diagnoses. This work represents a bridging of several different fields, including sequential decision-making, reinforcement learning, spatial statistics, and epidemiology, all in a Bayesian context. However, thus far, only two algorithms from these fields, TS and BYM, have been tested in simulation in the context of mobile HIV testing. The current simulation code allows for the addition of new strategies as modules on top of the larger evaluative framework; therefore, exploring additional algorithms can be easily undertaken in future work, which may allow us to identify new strategies that preserve simplicity of implementation and offer greater yields of new diagnoses.

Our study has several limitations. While we have expanded the number of zones in this paper to explore the performance of these algorithms beyond the small set of uncorrelated geographic locations in the earlier toy model, we have not yet included a temporal component to our analyses. Hotspots for detecting new cases of undetected HIV infection may shift, not only in space, but in time, both in the short-term (e.g., with opening and closing of social venues) and the longer term (e.g., as neighborhood demographics change). In addition, the ICAR prior in the BYM model requires an assumption about contiguous zones, namely that observations in immediate neighbors will be correlated [24]. However, this correlation by virtue of adjacency in the setting of HIV testing may not hold. For instance, a gay bar may exist in the context of a neighborhood that does not share the demographic characteristics of its patrons. This problem where geographic proximity exists among zones but the probability of finding undetected cases of HIV infection among them may be disparate can be addressed by spatial boundary detection methods, but a discussion of them is beyond the scope of this paper [25]. Finally, the simulation study results suggest that the choice of 10 unique zones for the initial learning strategy for the BYM strategy works well in comparison to TS under our specific HIV testing data settings. However, in future applications of the model, these choices may need to be revisited based on problem-specific prevalences and zonal geography.

Our portrayal of the epidemiology of HIV infection and the mechanics of HIV testing is, admittedly, simplistic. Among the many details that it omits are the use of testing services by people who already know their infection status; the possibility that infection risk may influence an individual’s decision to obtain an HIV test; the costs of moving a mobile testing facility from one location to another; more complicated forms of immigration and emigration, including daily travel between zones, via either public or private transportation, for work or other activities; and the possibility that even a few HIV tests on a single day might have a material influence on the prevalence of infection and the success of continued testing in a given zone on a given day. Each of these simplifications can be accommodated within the current analytic framework if circumstances suggest that they are more important than we have argued here.

## Conclusions

TS and the BYM algorithm both offer ways to manage the exploration–exploitation trade-off in deciding where to locate mobile HIV testing services from day to day. TS may be more suitable for settings where there are resource constraints in terms of computing power and statistical support. Spatial algorithms could be important tools, particularly if their execution could be simplified for use by non-experts in the field.

## Additional files

Additional file 1: **Algorithm 1** Thompson sampling strategy. **Algorithm 2** BYM strategy. **Algorithm 3** Clairvoyant strategy. (DOCX 28 kb)

Additional file 2: **Figure S1.** Example of grid of true underlying prevalences of undiagnosed HIV infection. **Figure S2.** Estimated prevalence of undiagnosed HIV infection by strategy at five time points. **Figure S3.** Cumulative visits to each zone by strategy at five time points. **Figure S4** Example of grid of true underlying prevalences of undiagnosed HIV infection. **Figure S5.** Estimated prevalence of undiagnosed HIV infection by strategy at five time points. **Figure S6.** Cumulative visits to each zone by strategy at five time points. (ZIP 3464 kb)

Additional file 3: **Table S1.** Results for main analysis and sensitivity analyses for Clairvoyance. (DOCX 13 kb)

## Abbreviations

BYM: Besag York Mollié
CAR: Conditional autoregressive
HIV: Human immunodeficiency virus
ICAR: Intrinsic conditional autoregressive
MCMC: Markov chain Monte Carlo
TS: Thompson sampling
