# Supplementary material for: Bayesian adaptive algorithms for locating HIV mobile testing services
Source: BMC Med. 2018 Sep 3;16:155. doi: 10.1186/s12916-018-1129-0 (PMC6120098; doi:10.1186/s12916-018-1129-0)
Supplement: Supplementary file 1 — Algorithm 1 Thompson sampling strategy. Algorithm 2 BYM strategy. Algorithm 3 Clairvoyant strategy. (DOCX 28 kb) [file 12916_2018_1129_MOESM1_ESM.docx]

**Additional file 1**

| **Algorithm 1** Thompson sampling Strategy |
| --- |
| For each zone *i = 1…,n^2^* set *X_i_(0)=0, Y_i_(0)=0*.  **for each** *t = 1, 2…t_max_,* **do**  For each zone *i = 1…,n^2^,* sample *θ_i_(t)* $\theta_{i}(t)$from the Beta *(α_i_ + X_i_(t), β_i_ + Y_i_(t))* distribution.  Select zone *j=argmax_i_ θ_i_(t).*  Perform *m* Bernoulli trials in zone *j* with success probability *UP_j_(t)* and observe *x_j_* successes and (*m-x_j_*) failures.  Let *X_j_(t + 1) = X_j_(t) + x_j_* and *Y_j_(t + 1) =Y_j_(t) + (m-x_j_).*  For all zones *i ≠ j,* let *X_i_(t + 1) = X_i_(t)* and *Y_i_(t + 1) =Y_i_(t).*  **end** |

| **Algorithm 2** BYM Strategy |
| --- |
| For each zone *i = 1, …, n^2^* set *X_i_(0)=0, Y_i_(0)=0*.  **do while the number of unique visited zones is < 10:**  For each zone *i = 1, 2, …,n^2^,* sample *θ_i_(t)* $\theta_{i}(t)$from the Beta *(α_i_ + X_i_(t), β_i_ + Y_i_(t))* distribution.  Select zone *j=argmax_i_ θ_i_(t).*  Perform *m* Bernoulli trials in zone *j* with success probability *UP_j_(t)* and observe *x_j_* successes and (*m-x_j_*) failures.  Let *X_j_(t + 1) = X_j_(t) + x_j_* and *Y_j_(t + 1) =Y_j_(t) + (m-x_j_).*  For all zones *i ≠ j,* let *X_i_(t + 1) = X_i_(t)* and *Y_i_(t + 1) =Y_i_(t).*  **do while the number of unique visited zones is ≥ 10:**  Fit the hierarchical Bayesian spatial logistic regression model:  $X_{i}\left( t \right)\vert p_{i},Y_{i}(t)\sim\mathrm{Binomial}\left( Y_{i}\left( t \right)+X_{i}(t), p_{i} \right);\mathrm{logit}\left( p_{i} \right)=\beta_{0}+\phi_{i}+\theta_{i}$  where $X_{i}\left( t \right)$ is the total number of identified HIV cases in zone *i* up to time *t*, $Y_{i}\left( t \right)+X_{i}(t)$ is the total number of administered tests in zone *i* up to time *t*, $p_{i}$ represents the actual but unobserved prevalence at zone *i*, $\beta_{0}$ is the shared intercept term, $\phi_{i}$ is the spatial random effect which follows the ICAR distribution, and $\theta_{i}$ is the exchangeable random effect (normally distributed with constant variance parameter). Note that only previously sampled zones contribute data to the fitting of this model.  After fitting the model, we obtain posterior samples from $f(p_{i}\vert\boldsymbol{X}\left( t \right), \boldsymbol{Y}(t))$ for each zone (even those that haven’t been visited yet) where $\boldsymbol{X}(t)$ and $\boldsymbol{Y}(t)$ are the complete set of data from all currently sampled zones ($\boldsymbol{X}\left( t \right)=\left( X_{1}\left( t \right),\ldots,X_{n^{2}}\left( t \right) \right)^{T}$; $\boldsymbol{Y}(t)$ defined similarly). We then randomly select a value from each distribution, *θ_i_(t)*.  Select zone *j=argmax_i_ θ_i_(t).*  Perform *m* Bernoulli trials in zone *j* with success probability *UP_j_(t)* and observe *x_j_* successes and (*m-x_j_*) failures.  Let *X_j_(t + 1) = X_j_(t) + x_j_* and *Y_j_(t + 1) =Y_j_(t) + (m-x_j_).*  For all zones *i ≠ j,* let *X_i_(t + 1) = X_i_(t)* and *Y_i_(t + 1) =Y_i_(t).*  **End** |
| Prior Specifications:   - $\beta_{0}\sim Normal(0, 2.85)$; Results in $\approx$ *uniform*(0,1) prior probabilities for each zone a priori assuming no excess variability in the data. - $\sigma_{\phi}^{2}$ (variance parameter for the ICAR random effect) $\sim inverse-gamma(3.00, 2.00)$ - $\sigma_{\theta}^{2}$ (variance parameter for the exchangeable random effect) $\sim inverse-gamma(3.00, 2.00)$ |

| **Algorithm 3** Clairvoyant Strategy |
| --- |
| **For each** *t = 1, 2…t_max_* **do**  Select zone *j= argmax_i_ UP_i_(t)*  Perform *m* Bernoulli trials with success probability *UP_j_(t)*.  **end** |
