# Supplementary material for: Bayesian adaptive algorithms for locating HIV mobile testing services
Source: BMC Med. 2018 Sep 3;16:155. doi: 10.1186/s12916-018-1129-0 (PMC6120098; doi:10.1186/s12916-018-1129-0)
Supplement: Supplementary file 2 — Figure S1. Example of grid of true underlying prevalences of undiagnosed HIV infection. Figure S2. Estimated prevalence of undiagnosed HIV infection by strategy at five time points. Figure S3. Cumulative visits to each zone by strategy at five time points. Figure S4 Example of grid of true underlying prevalences of undiagnosed HIV infection. Figure S5. Estimated prevalence of undiagnosed HIV infection by strategy at five time points. Figure S6. Cumulative visits to each zone by strategy at five time points. (ZIP 3464 kb) [file 12916_2018_1129_MOESM2_ESM.zip › Additional File Figure S1R1.pptx]

## Slide 1
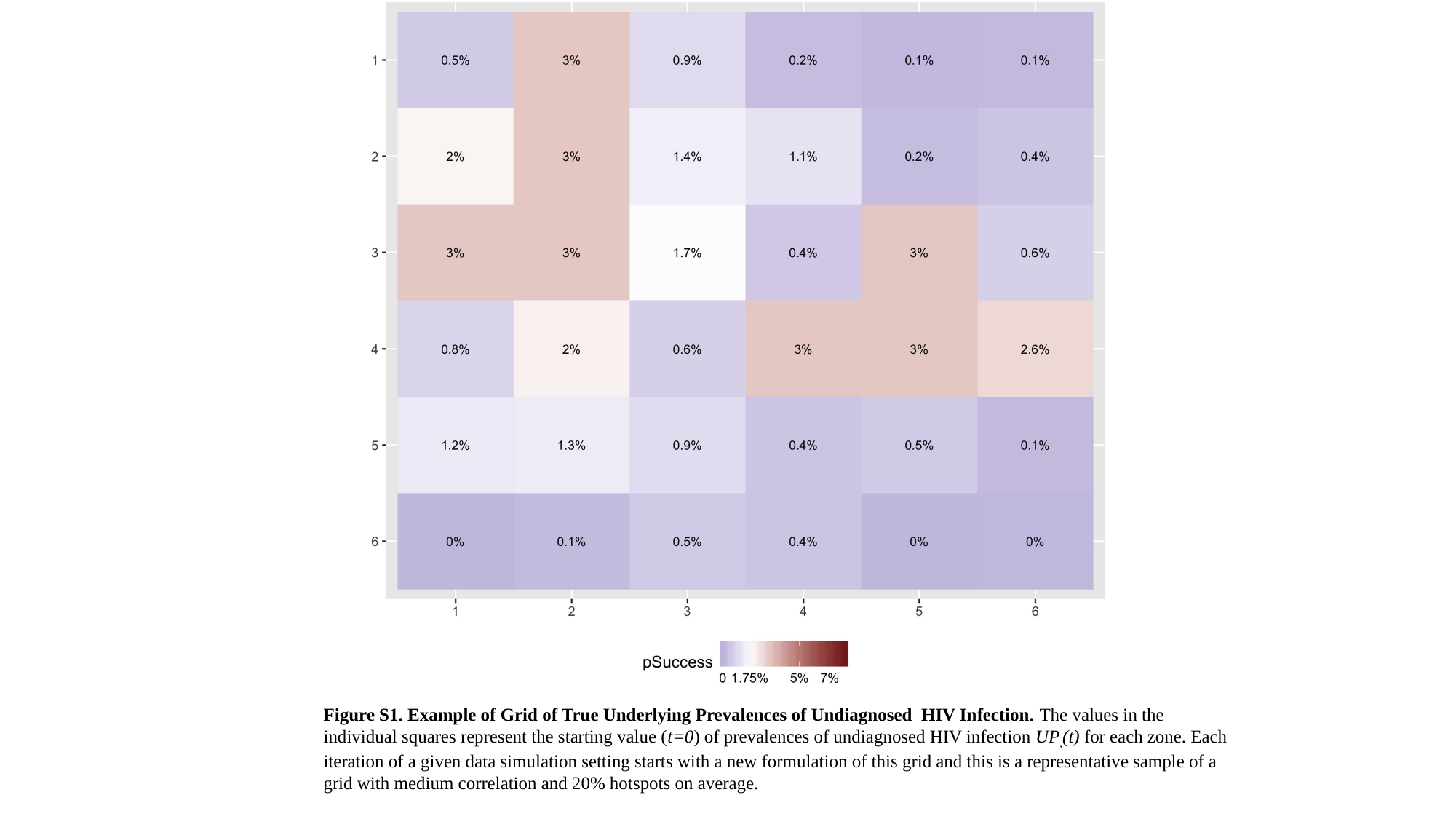

Figure S1. Example of Grid of True Underlying Prevalences of Undiagnosed HIV Infection. The values in the individual squares represent the starting value (t=0) of prevalences of undiagnosed HIV infection UP,(t) for each zone. Each iteration of a given data simulation setting starts with a new formulation of this grid and this is a representative sample of a grid with medium correlation and 20% hotspots on average.
