# Supplementary material for: Bayesian adaptive algorithms for locating HIV mobile testing services
Source: BMC Med. 2018 Sep 3;16:155. doi: 10.1186/s12916-018-1129-0 (PMC6120098; doi:10.1186/s12916-018-1129-0)
Supplement: Supplementary file 2 — Figure S1. Example of grid of true underlying prevalences of undiagnosed HIV infection. Figure S2. Estimated prevalence of undiagnosed HIV infection by strategy at five time points. Figure S3. Cumulative visits to each zone by strategy at five time points. Figure S4 Example of grid of true underlying prevalences of undiagnosed HIV infection. Figure S5. Estimated prevalence of undiagnosed HIV infection by strategy at five time points. Figure S6. Cumulative visits to each zone by strategy at five time points. (ZIP 3464 kb) [file 12916_2018_1129_MOESM2_ESM.zip › Additional File Figure S2R1.pptx]

## Slide 1
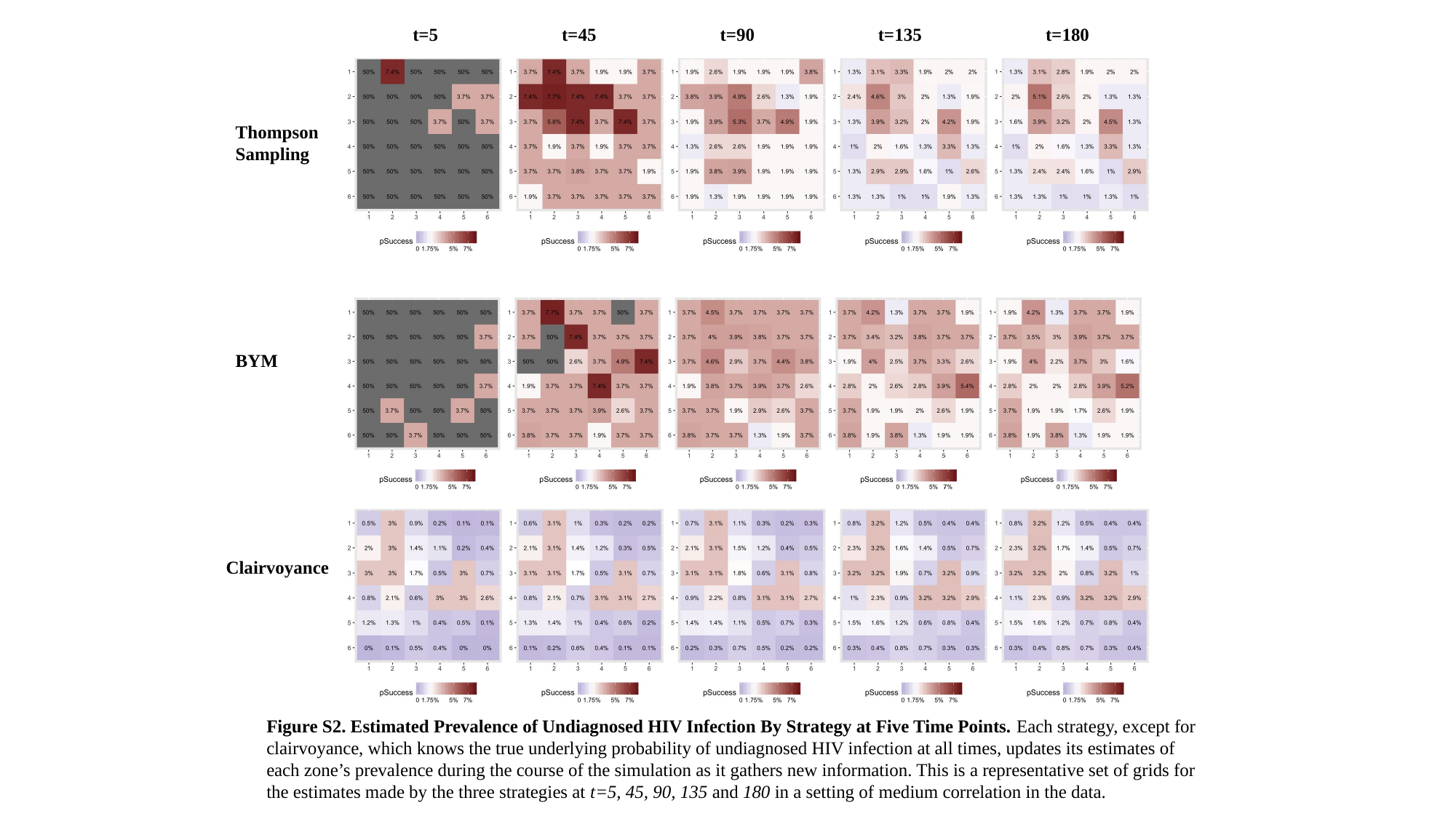

t=5
t=45
t=90
t=135
t=180
Thompson Sampling
BYM
Clairvoyance
Figure S2. Estimated Prevalence of Undiagnosed HIV Infection By Strategy at Five Time Points. Each strategy, except for clairvoyance, which knows the true underlying probability of undiagnosed HIV infection at all times, updates its estimates of each zone’s prevalence during the course of the simulation as it gathers new information. This is a representative set of grids for the estimates made by the three strategies at t=5, 45, 90, 135 and 180 in a setting of medium correlation in the data.
