# Supplementary material for: Bayesian adaptive algorithms for locating HIV mobile testing services
Source: BMC Med. 2018 Sep 3;16:155. doi: 10.1186/s12916-018-1129-0 (PMC6120098; doi:10.1186/s12916-018-1129-0)
Supplement: Supplementary file 2 — Figure S1. Example of grid of true underlying prevalences of undiagnosed HIV infection. Figure S2. Estimated prevalence of undiagnosed HIV infection by strategy at five time points. Figure S3. Cumulative visits to each zone by strategy at five time points. Figure S4 Example of grid of true underlying prevalences of undiagnosed HIV infection. Figure S5. Estimated prevalence of undiagnosed HIV infection by strategy at five time points. Figure S6. Cumulative visits to each zone by strategy at five time points. (ZIP 3464 kb) [file 12916_2018_1129_MOESM2_ESM.zip › Additional File Figure S6R1.pptx]

## Slide 1
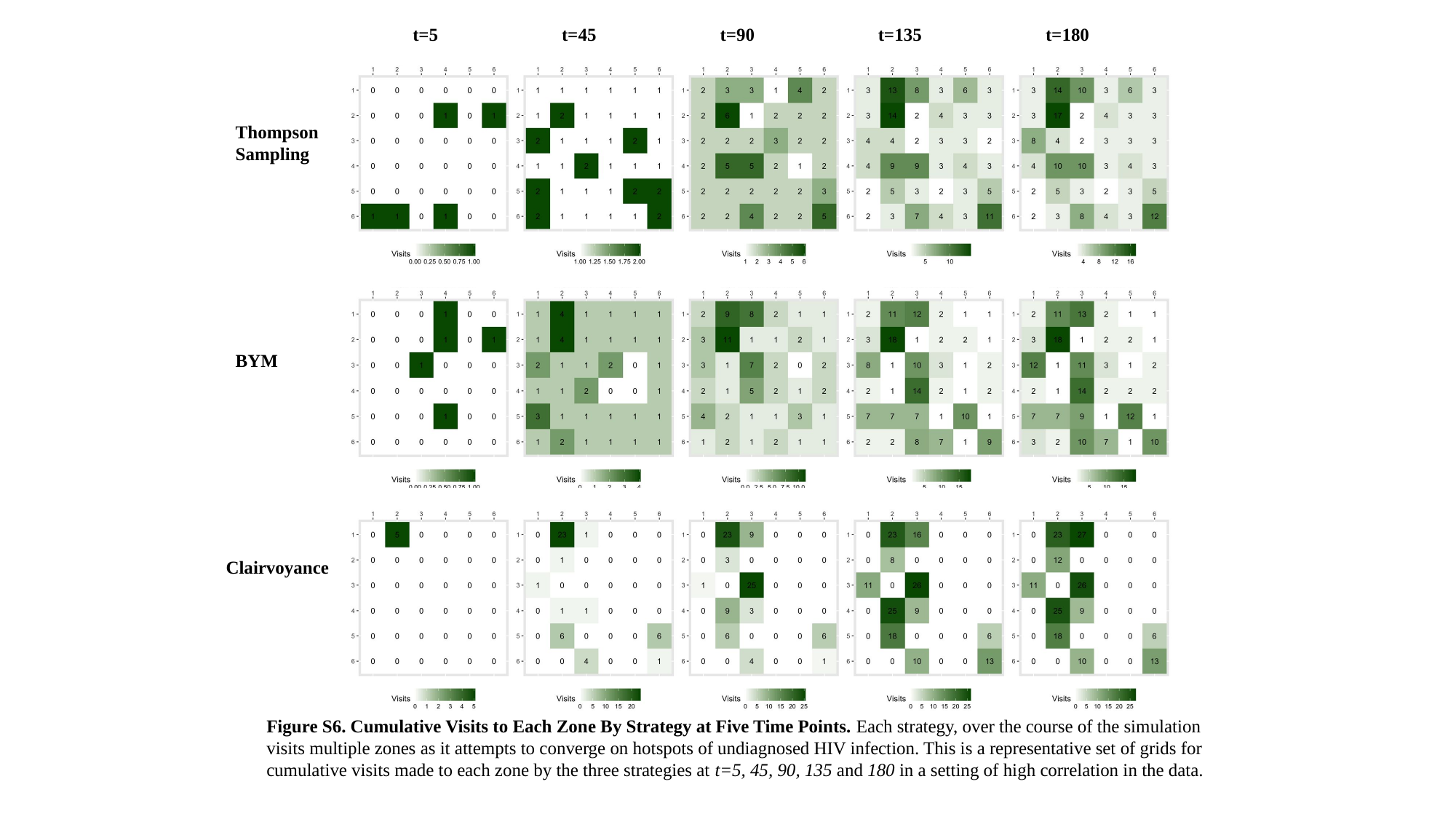

t=5
t=45
t=90
t=135
t=180
Thompson Sampling
BYM
Clairvoyance
Figure S6. Cumulative Visits to Each Zone By Strategy at Five Time Points. Each strategy, over the course of the simulation visits multiple zones as it attempts to converge on hotspots of undiagnosed HIV infection. This is a representative set of grids for cumulative visits made to each zone by the three strategies at t=5, 45, 90, 135 and 180 in a setting of high correlation in the data.
