# Supplementary material for: Bayesian adaptive algorithms for locating HIV mobile testing services
Source: BMC Med. 2018 Sep 3;16:155. doi: 10.1186/s12916-018-1129-0 (PMC6120098; doi:10.1186/s12916-018-1129-0)
Supplement: Supplementary file 3 — Table S1. Results for main analysis and sensitivity analyses for Clairvoyance. (DOCX 13 kb) [file 12916_2018_1129_MOESM3_ESM.docx]

| **Table S1. Results for Main Analysis and Sensitivity Analyses for Clairvoyance** | | | |
| --- | --- | --- | --- |
|  |  | **Clairvoyance** | |
| **Parameters** | **Values** | **New Diagnoses** | **New Diagnoses** |
|  |  | **Mean** | **Std Dev** |
| Main Analysis | N/A | 141.87 | 11.83 |
| Grid Dimensions | 4 x 4 | 139.28 | 11.78 |
| Grid Dimensions | 5 x 5 | 139.70 | 12.02 |
| Grid Dimension | 10 x 10 | 141.02 | 11.14 |
| % of New HIV-Negative Arrivals (times zone population divided 365 days) | 0 | 142.15 | 10.96 |
| % of New HIV-Negative Arrivals (times zone population divided 365 days) | 1.70% | 142.05 | 11.47 |
| % of New HIV-Negative Arrivals (times zone population divided 365 days) | 6.80% | 140.43 | 11.60 |
| % of New Infections (times zone population divided by 365 days) | 0 | 134.45 | 11.53 |
| % of New Infections (times zone population divided by 365 days) | 0.33% | 138.56 | 10.98 |
| % of New Infections (times zone population divided by 365 days) | 1.32% | 147.58 | 11.46 |
| no-arrivals-or-infections | N/A | 141.87 | 11.83 |
| Level of Correlation, % of Hotspots in Grid | None, 20% (on average) | 142.02 | 11.26 |
| Level of Correlation, % of Hotspots in Grid | Low, 10% (on average) | 139.45 | 12.08 |
| Level of Correlation, % of Hotspots in Grid | Low, 30% (on average) | 142.02 | 11.51 |
| Level of Correlation, % of Hotspots in Grid | Medium, 20% (on average) | 141.41 | 11.46 |
| Level of Correlation, % of Hotspots in Grid | High, 20% (on average) | 142.00 | 11.58 |
| Days Until Return to Unobserved, Uninfected Pool | 10 | 141.06 | 11.53 |
| Days Until Return to Unobserved, Uninfected Pool | 90 | 141.86 | 11.67 |
| Days of Testing | 90 | 69.42 | 7.94 |
| Days of Testing | 365 | 296.32 | 18.05 |
| Tests Per Day | 10 | 56.32 | 7.57 |
| Tests Per Day | 40 | 225.97 | 14.96 |
